# A qualitative study of assessing learning needs and digital health literacy in pregnancy: Baby Buddy Forward Greek findings

**DOI:** 10.18332/ejm/150770

**Published:** 2022-09-01

**Authors:** Kleanthi Gourounti, Antigoni Sarantaki, Maria-Eleni Dafnou, Eleni Hadjigeorgiou, Aikaterini Lykeridou, Nicos Middleton

**Affiliations:** 1 Department of Midwifery, School of Health and Care Sciences, University of West Attica, Athens, Greece; 2 Department of Nursing, Faculty of Health Sciences, Cyprus University of Technology, Limassol, Cyprus

**Keywords:** learning needs, digital health literacy, pregnancy, information seeking, qualitative study

## Abstract

**INTRODUCTION:**

The purpose of this study was to explore the learning needs and the digital health literacy of pregnant women in Greece regarding perinatal health and care issues.

**METHODS:**

This was a qualitative study involving thirteen Greek pregnant women, in two focus groups of primiparous and multiparous. The interview topic guide was developed by consensus during a training workshop of the European Baby Buddy Forward research program. The interviews were recorded, transcribed and inductively content analyzed.

**RESULTS:**

Pregnant women described a range of learning needs and identify antenatal classes, health professionals, Internet, books, friends and relatives, as their main sources of information. Women expressed satisfaction in terms of their communication with midwives, but they expressed ambiguity regarding communication with doctors. With regard to the Internet, women highlighted their concern about the validity of information and point out the difficulties they face in order to access reliable scientific resources. Moreover, the process of seeking information online occurs in parallel and independently from healthcare providers, who discourage it, thus, many women were reluctant to discuss any information they retrieved from the Internet with health professionals.

**CONCLUSIONS:**

The content of antenatal classes should be tailored to address pregnant women learning needs more holistically. However, taking into consideration that a major source of information for pregnant women is the Internet, it is vital for health professionals to acknowledge this reality and provide pregnant women with trusted websites. It is also particularly important for health professionals to practice their communication skills and update their digital knowledge.

## INTRODUCTION

Pregnancy is one of the most sensitive and important stages of a woman’s life and may be a woman’s first contact with the healthcare system. Therefore, entering this multifaceted system for the first time, learning new information and following guidelines can be complicated, even for women with sufficient literacy skills, while for women with lower literacy skills, the experience can be significantly more demanding^1^. Since women’s health literacy is vital to improving the health of children and their families, women have been identified as a primary population for improving health literacy skills^2^.

Health literacy (HL) encompasses the knowledge, motivation and competencies of accessing, understanding, appraising and applying health-related information within healthcare, disease prevention and health promotion settings. Pregnant women represent a population where there is a growing need for adequate HL levels due to the abundance of health information they receive throughout this period^1^. A recent systematic review by Ghiasi^3^ shows that the majority of pregnant women seek information from health professionals, followed by informal source (family and friends) but almost always from the Internet as well. Maternal health literacy is the key to achieving a healthy pregnancy but it can also affect pregnancy outcomes through improving the quality of communication with healthcare providers and hence the quality of the healthcare received^4^.

Telehealth is broadly defined as the use of electronic information and telecommunications technologies to support healthcare, including health education and population health management^5^. Mobile apps, frequently called mHealth applications, may offer versatility and personalization, and may include features that promote ease of practice, use spoken and written language, use multiple languages, can be scripted at a level that addresses the needs of users with low literacy and numeracy skills, and may be viewed as often as needed by a patient^6-8^. Mobile health (mHealth) apps have gained noteworthy popularity over the last few years due to their tremendous benefits, such as lowering healthcare costs and increasing patient awareness. Rather than being a passive participant, digital solutions provide the opportunity for the individual to be an active participant in their health.

Mobile apps supporting interactive education have the potential to greatly increase interest, because the user actively participates in the learning process^9^. Limited computer experience and low health literacy, which are more prevalent in individuals from low socioeconomic strata, do not appear to impact their ability to use interactive health education programs effectively^6^. Knowledge and empowerment through enhanced informational and emotional support can support active participation in the care and improve decision-making skills.

However, the majority of pregnant women are worried about the validity of information on the Internet and apps. While there are many sources of information, the quality of information is often problematic^10^. Misinformation or incorrect information can make pregnant women anxious about the information they encounter^11^. Few pregnancy apps have undergone scrutiny to evaluate the accuracy of the information they provide and their alignment with current obstetric guidelines^12^. Yet, paradoxically, research studies have shown that women tend not to discuss the information they received via the Internet with health professionals, often due to the reluctance of healthcare providers themselves to engage in conversation^12-15^. Taking that into account, many researchers have suggested the need for healthcare providers to take a more active role in directing pregnant women to valid and reliable web resources^12-14,16-22^.

To the best of our knowledge this is the first qualitative research study on information-seeking practices and on learning needs of pregnant women in Greece. The purpose of the study was to gain an understanding of pregnant women’s learning needs about maternal health issues, sources of information and satisfaction with informational support. Furthermore, it was aimed to explore pregnant women’s views in terms of the use of the Internet as a source of information during pregnancy and their experience with regard to accessing and appraising the validity of information they receive online.

## METHODS

### Design

For the purpose of this study, a qualitative descriptive design was employed. Two focus groups with nulliparous and multiparous pregnant women were conducted from January to February 2019 in the Department of Midwifery in University of West Attica, Athens.

### Participants, recruitment and data collection process

Participants in the present study were 13 Greek pregnant women selected through a purposive sampling method. In order to achieve maximum variation, the sampling strategy considered parity, age, education level, employment status and gestational age. In addition, a demonstrated ability to express their experiences and opinions vividly was considered at the sampling stage. Women included were Greek speaking adults with no medical condition. Interested participants were recruited through prenatal clinics, healthcare centers and midwife or obstetrician offices. The authors did not have a relationship with the participants or recruitment center and were not involved in the direct care of the participants. The participants were contacted in advance, via e-mail and/or telephone call by a member of the research team in order to provide explanations about the purpose of the study and make arrangements for the meeting at a date and time most convenient for all group participants. A friendly and interference-free place was chosen for the meetings on location at the Department of Midwifery. Before the beginning of the session, there was time to discuss the purpose of the study and provide more information. Participation was voluntary and a signed consent form was provided by all women who participated in the sessions. Participants were assured that all the information and recording would be confidential and would be used only for the purpose of this study. Anonymity was ensured when presenting quotes for the data analysis. During the discussion, all participants were highly encouraged to express their opinions and ideas, and to protest where they thought was necessary. The study protocol was granted approval by the Cyprus National Bioethics Committee (Reference number: EEBK EΠ 2018.01.124).

### Data collection

Semi-structured interviews and field notes were used to collect data. An interview topic guide developed by consensus during a training workshop of the European Baby Buddy Forward research program was used to structure the discussion. The first focus group (FG1) consisted of 8 multiparous women (FG1 pregnant women: P1–P8) and the second group (FG2) consisted of 5 primiparous women (FG2 pregnant women: P1–P5). The pregnant women in both groups found adequate opportunities to participate in the conversation. Participants had extensive experiences to share with the group and the duration of the sessions were adequate to explore the topic in greater depth. Interviews started by posing a general question and the interview topic guide consisted of six open questions (Table 1). The open questions were about the learning needs during pregnancy, the sources of information, the experiences regarding access to the information, the use of the Internet, the interactions between women and health professionals, and satisfaction with informational support in relation to being able to make informed decisions during pregnancy. Follow-up prompts and probe questions were used as necessary. An experienced moderator and an observer (both academic midwives) ran the sessions. Women were actively encouraged to express their needs, worries, ideas and opinions by the moderator (second author). The first author documented observations of the non-verbal reactions of participants during the interview in a field note diary. The sessions lasted between 60 and 90 minutes and were audio recorded. The discussion continued until data saturation was reached, i.e. when there was no new information added to the existing data.

**Table 1:** Questions of the interview guide used during the two focus groups

*Opening questions*	*Follow-up questions*
1. What kind of information do you need during pregnancy? For example, it can be about pregnancy, labor and/or the care of the newborn.	Do you feel you are satisfied with the information that you have received? Is there something you want to learn more about?
2. What is your opinion about birth preparation and parenthood classes provided by midwives?	Have you ever attended any such course and/or class. Why yes/Why not?
3. How would you describe your experience at an appointment with healthcare providers (midwives, pediatricians, gynecologists)?	Did you have the opportunity to ask your doctor/midwife questions? How was the communication and interaction? Can you express your worries to your doctor or your midwife? You feel the need for more information about something?
4. Other than health professional (obstetrician, midwives) and birth preparation classes, which are the main sources of information for you during pregnancy?	For example, do you think that the Internet, television or friends are a source of information?
5. Which is your experience from searching on the Internet and/or using an app?	What do you search on the Internet usually? What information would you want an application to have? How you evaluate the validity of the information?
6. If you find a piece of information on the Internet, do you discuss it with your doctor or midwife?	Do you feel like your doctor or your midwife encourage or discourage you to seek information on the Internet?
**Exit question**	
Is there anything additional you would like to say about the information you got during pregnancy?	
**Probe questions**	
Can you please say more about this? Can you give an example of that? Can you tell me something else about that?	

### Statistical analysis

The digital audio recordings were transcribed verbatim. The transcripts were read multiple times and independently by the authors for the purpose of familiarization with the data. Data analysis was conducted using the conventional content analysis method which is an appropriate method for offering rich and compelling insights within health research^23^. Coding of the material was performed manually in an iterative process and organization into categories and themes was agreed by consensus of the research team. Researchers identified patterns by extracting sentences and phrases that were coded. The codes were merged by two independent investigators (first and third author) and related codes were classified in subtheme categories. Categories (subthemes) were organized in themes with meaningful essence. Portions of the data were gathered by each theme by identifying words and expressions related to the concept or to specific survey questions which reflected the underlying theme. In order to demonstrate the theme components, selected quotes from the interviews are presented.

## RESULTS

Thirteen women participated in the two focus groups (8 nulliparous and 5 multiparous). The mean age of the participants was 31 years (range: 22–43), nine women were employed and four were not working, six women were lyceum graduates, five women had a Bachelor’s degree and two women had a Master’s degree. Data analysis identified eight subthemes and three main themes. The three main themes were the following: ‘learning needs during perinatal period’, ‘sources of information’, and ‘challenges to accessing information during perinatal period’. The eight subthemes were as follows: ‘interactions between women and health care professionals’, ‘difficulty to distinguish between reliable and unreliable information’, ‘learning needs about pregnancy-related common complains, disorders and examinations’, ‘learning needs about breastfeeding, infant care and development’, ‘health professionals and antenatal classes as a source of information’, ‘traditional sources of information’, and ‘the use of the Internet as source of information’ (Table 2).

**Table 2:** Examples of the analysis process, codes, subtheme categories and themes

*Example codes*	*Subtheme categories*	*Themes*
Pregnancy-related common complains Abnormal symptoms during pregnancy Medical examinations Exercise and nutrition during pregnancy Coping with labor	Learning needs about mental and physical health, lifestyle and healthcare during pregnancy	Learning needs during perinatal period
Breastfeeding establishment Introduction of solid food Neonatal care and baby’s development and psychology Psychological issues in pregnancy and postnatal period Sibling preparation for coming of new baby	Learning needs about breastfeeding, neonatal care and baby development	
Discussion with a midwife Antenatal classes as valuable source of information	Health professionals and antenatal classes as a source of information	Sources of information during pregnancy
Information from websites of well-known and recognizable organizations	Internet as source of information	
Books Friends and family	Traditional sources of information	
Making questions and expressing worries to midwives that made women feel comfortable Uncomfortable and restricted communication with medical doctors	Interactions with health professionals	Challenges to accessing and appraising information during perinatal period
Preference to get information from well-known websites of recognizable organization	Difficulty to distinguish between reliable and unreliable information	

### Learning needs during perinatal period

Women were asked to indicate the topics that they would like to receive more information about during pregnancy period. Two subthemes emerged: ‘learning needs about mental and physical health, lifestyle and healthcare during pregnancy’ and ‘learning needs about breastfeeding, infant care and development’.

*Learning needs about mental and physical health, lifestyle and healthcare during pregnancy*

As was noted mostly by the group of primiparous pregnant women, they would like to receive more information about the antenatal period. Among the learning needs frequently noted by the primiparous women were: pregnancy-related common complains, abnormal symptoms during pregnancy, medical examinations, exercise and nutrition during pregnancy, coping with labor, psychological adjustment in pregnancy, and breastfeeding establishment:

*‘At the beginning of my pregnancy I was mostly concerned about medical issues, and more specifically about what I could know in order to be sure that the fetus was fine. After the seventh month of my pregnancy I became more concerned for the labor, the physiology of the labor, and the decisions I need to make during the process of the delivery … and from now on, I became more interested to know more about the care of the baby.’* (FG2, P1)

*‘When I got pregnant, I was mostly thinking the tests and exams that I had to do. Now, that I am running the eighth month of my pregnancy I am thinking how I will cope with the labor and the breastfeeding.’* (FG2, P4)

*Learning needs about breastfeeding, neonatal care and baby development*

Mostly the group of multiparous women referred that they would like to receive more information about the postnatal period. Among the learning needs frequently noted by the multiparous women were: breastfeeding establishment, introduction of solid food, neonatal care and baby’s development and psychology, psychological issues in pregnancy and postnatal period, and sibling preparation for coming of new baby.

### Sources of information

Women were asked to indicate the sources from which they received information during pregnancy. Three subcategories emerged: ‘health professionals and antenatal classes as a source of information’, ‘personal sources of information’, and ‘Internet as source of information’.

*Health professionals and antenatal classes as a source of information*

Almost all pregnant women reported that the most frequent source of information was ‘discussion with a midwife’. They reported that they usually had a verbal communication with a midwife either as part of antenatal classes or through personal meetings. Almost all participants from both focus groups considered the participation in antenatal classes as an extremely valuable source of information. They reported that through the antenatal classes they received reliable information. Some, nevertheless, suggested that they wanted the antenatal classes to be more personal and the information to be more up-to-date. More importantly, women felt that antenatal classes provided a time and space to develop an emotional bonding with the midwives, develop a relationship with other pregnant women, cope with their emotional difficulties and expelled fear about birth and other fearful things. Both primiparous and multiparous women had a similar attitude towards the value of antenatal classes. The majority of participants reported that they usually obtained information from obstetricians and pediatricians. Some of the pregnant women said that, during the antenatal classes, they had more time to express their worries and their questions, rather than during an appointment with the obstetrician.

*‘Certainly, you get a lot of information from a book, from the Internet and even from an application. However, interpersonal contact with your midwife or your doctor cannot be replaced … these [re: antenatal classes] are very useful because you are provided with well-documented information. And your pregnancy is something very new in your life, so I find classes quite helpful and necessary.’* (FG2, P2)

*‘Antenatal classes are valuable in each and every pregnancy, this means you don't just do the classes during the first child and you won't need them ever again.’* (FG1, P5)

*Internet as source of information*

Almost all pregnant women mentioned that they were seeking information on the Internet about the pregnancy (e.g. medical exams and body changes during pregnancy). The majority reported that they tend to use the Internet for minor issues but for more serious issues they preferred to seek information directly from their doctor or midwife. Pregnant woman noted that they prefer to get information from websites of well-known and recognizable organizations. Many of them expressed their concern about the trustworthiness of online sources of information and reported that they did not trust the information on mothers’ blogs. Nevertheless, they did identify that personal experience is important in addition to the information support they receive from healthcare providers:

*‘I have been informed by the midwives, doctors, obstetricians, and pediatricians and sometimes I go to the Internet for information … and based on personal experience I think slowly and by yourself you start and learn in practice.’* (FG1, P3)

*‘I usually trust and check sites of well-known organizations. However, I don't trust mother's blogs.’* (FG1, P5)

Furthermore, some women reported that they preferred to read medical articles from personal websites of healthcare professionals and that they like to first check the credentials of the author of the article as well as how current it is:

*‘If I read an article by a doctor that was published in 2010, I will be worried about it and I will keep searching for further more recent information.’* (FG2, P3)

*‘Usually when I read a website that belongs to a doctor or to a midwife, I try to check his/her CV and previous experience for understanding the credibility of site.’* (FG1, P2)

*Personal sources of information*

Some participants reported that they received information through books. A few of them added that they received information from ‘trustworthy’ friends and family. However, many pregnant women expressed concern about receiving information from their mothers and their mothers in law. They attributed this concern to the existence of a generation gap between them and felt that peers who share philosophies towards pregnancy and baby’s care are a preferred source of information:

*‘When I gave birth, my mom felt incapable of giving me any advice ... it was as my mother had not raised two children!!’* (FG2, P4)

*‘There are some people [re: friends] who you trust and are important to you and you respect them, and you feel that you can ask them some of your questions.’* (FG2, P3)

*‘I find it very important that there are people [re: friends] around us who we can trust and share the same life philosophy with us, that is important.’* (FG1, P8)

### Challenges to access information during perinatal period

Women were asked to indicate the challenges that they faced while accessing and appraising information during pregnancy. Two subthemes emerged: ‘interactions between women and health care professionals’, and ‘difficulty to distinguish between reliable and unreliable information’.

*Interactions with health professionals*

As noted by the participating women, they considered the information provided by the midwives, the obstetricians and the pediatricians as reliable and trusted. They considered their interaction with the midwife satisfying. They felt confident to ask midwives questions and to express their worries, since midwives were considered calm and not dogmatic. Many considered that their communication and their interactions with medical doctors was not equally satisfying and attributed this mainly to doctors’ time restrictions and lack of personal relationship, but often also to doctors’ distant attitude. Women described that they often feel uncomfortable with doctors and are reluctant to pose further questions or express worries and opinions, because they are often met with indifference or find their response unhelpful and uninformative:

*‘The problem for me again is the pediatrician … I have not only changed two but I have changed four to five, indeed everyone expresses different views and opinions and you really start asking yourself what did he tell me, why did he tell me this, should I look for something else, something more, and then the Internet will it confused me more?’* (FG1, P2)

*‘… I just do not feel the same comfort as getting to ask the midwife because there is a personal contact there. That's what I think is changing, for some were just another client … I have not felt a denial, or unwillingness to answer, I just have not felt this comfort …’* (FG1, P6)

Participating women reported that they usually avoid discussing with their doctors or their midwives, information they obtained from the Internet. Women mentioned that they would discuss such information only if they considered the source as extremely reliable. After all, many felt that healthcare providers are negatively predisposed against the Internet and thus they were not only reluctant to engage in discussion about a piece of information they obtained from the Internet but also actively discouraged them to searching on the Internet:

*‘They do not like us searching on the Internet, it might contradict their knowledge.’* (FG1, P3)

*‘They generally do not want us to interfere with their work, it is as if we are questioning them, we are not allowed to get into their territory.’* (FG2, P2)

*Difficulty to distinguish between reliable and unreliable information*

Women were worried about the reliability of the information on websites and other digital sources. One pregnant woman said: *‘it's a bit difficult to find reliable articles, you have to know in advance a specific source to look for articles that are scientific and reliable’*. Many of them expressed the preference to get information from websites of well-known and recognizable organizations. A number of women stated that they prefer to read medical articles from the personal websites of healthcare professionals and, first, they would look for the credentials by checking the Curriculum Vitae of the authors. One pregnant woman mentioned that the date of publication is also important. The majority of the participating woman were reluctant to use mothers’ forums or blogs, because they found the information not reliable. Only one woman mentioned that she reads information posted on mothers’ blogs, but she is trying to find the average opinion. Lastly, a number of pregnant women suggested that it will be a good idea for midwives and other healthcare providers to provide them with a list of specific scientific sites that they can consult:

*‘But it's not that easy when typing something, unfortunately the first options that google will throw at you is from a forum that mothers talk about. You cannot easily find scientific articles, you need to know specific databases to look for. This is a problem. Although I visit mom's blogs, I don't consider them reliable, usually what I am doing is to look for the average opinion.’* (FG1, P4)

When presented with the idea of a pregnancy-related application in the Greek language, participating women found it appealing and referred that this would be considered reliable by them. They would like to see a variety of different topics covered in such an app and they wanted the information to be simple, concise and easy to understand. They also found the concept of including videos appealing and expressed the opinion that information should be based on the Hellenic healthcare system and culture:

*‘So, it will be something good if you could have a reliable tool to use even at 4 o'clock in the night.’* (FG1, P4)

## DISCUSSION

This qualitative study provided information about the learning needs about maternal health issues, sources of information and satisfaction with informational support of pregnant women in Greece. Our study findings demonstrate that multiparous pregnant women are more worried about the postnatal period, the development and care of the neonatal, while the nulliparous pregnant women seemed to be more worried about the antenatal period, as they have not experienced birth before and they do not know what to expect at labor. Our study findings are in accordance with findings of previous studies from different countries^3,22^. In terms of sources of information, it seems that the preferred source for Greek pregnant women is personal communication with healthcare professionals (midwives, obstetricians and pediatricians) either during routine appointments or at birth preparation classes with midwives. While the Internet is also a major source of information, pregnant women are aware of issues with the quality of information they read online. Moreover, based on our findings, the process of seeking information online seems to occur in parallel and independently from healthcare professionals, who discourage it, and thus women of our study stated that they are reluctant to discuss information they retrieved from the Internet with health professionals. Informal sources (such as friends and family) and own personal experience also appear important, yet they do not seem to question the trustworthiness of such information to the same extent.

The available literature and other study reports suggest that antenatal education provides women with necessary information about various aspects during the antenatal and postnatal period^24^. It seems that antenatal education improves women’s health during pregnancy, decreases the risk for complications, and increases the satisfaction during delivery^4,24^. Our responders emphasized that they found birth preparation courses useful, practical and informative. After the sessions, their fears were diminished and they felt confident and assured. As they mentioned, antenatal classes provide the time and space to express worries and thoughts, but also form a personal bond with each other and with midwives. Taking into consideration the impact that those courses have on pregnant women, National Health System of Greece should encourage community midwives to expand their professional knowledge-base and provide birth preparation classes to pregnant women in their community.

As for the quality of relationship they have with healthcare professionals, pregnant women felt that they had an excellent communication with their midwives, and that a midwife was the first person that came into their minds if they had anything to ask. This finding is in line with a study from Australia that claimed that women described discussions with a midwife as the most useful and common source of information^22^. Many of the women that participated in this study did not consider that this was also true in the case of the doctors. As they mentioned, they are more reluctant to express their worries to the doctor. Furthermore, pregnant women emphasized their disappointment that often doctors have different opinions and there is no common line. One study has demonstrated that women who received their care only from doctors reported that the Internet, rather than the doctor, was their most useful source of information^22^. It is worthy to mentioned that several other studies in the literature, report the lack of informational support during user-provider exchanges^3,17,21,25^. This study identifies the need for health professional to develop their communication skills in order to ensure that pregnant women feel confident to express themselves and participate actively in the care. Moreover, health professionals should follow guidelines and they should update their knowledge with the latest resources.

It is also interesting to note that in this study, it was not only nulliparous women who felt the need for informational support. Multiparous pregnant women, irrespective of previous experience, also seek information as many do not feel secure to make decisions when needed. They expressed that they needed more information about the baby’s care and development, as well as more information about vaccination, risk of chocking, co-sleeping, the duration of breastfeeding, the role of the father, to mention a few. Hence, birth preparation classes should not only focus on the physiology of pregnancy and birth, but should provide information about the postnatal period and multiparous women, and fathers should be actively encouraged to participate.

As expected, the use of Internet sources of information appears prevalent among Greek pregnant women. They are most likely to search about changes to their body, mandatory exams during the pregnancy, breastfeeding, nutrition, solid food and baby’s care and development. For medical issues, they prefer to consult their healthcare professionals (mainly for the serious matters). Our study findings, regarding the publicity of the Internet as the most common source of information, are very similar to other studies that have been reported^10,12,14,16,17,26,27^. It has also been reported that pregnant women are concerned about the validity of the information they get through the Internet. This finding is line with previous studies that identified the varied quality of pregnancy-related information on the Internet and apps, as well as how insecure women felt about it^3,10,16^. Furthermore, it is important to highlight that the majority of women in this study reported that, even though they were unsure about the quality of the information they found online, they avoided discussing any information they obtained online with healthcare professionals since they felt that they will be discouraged and criticized. Previous studies have also described this paradox^12-15^. Moreover, some of the participants suggested that midwives and other healthcare professionals should be in the position to suggest specific sites and other digital resources that they can trust, which has also been described previously^20,21,27^.

With regard to the prospect of an application in their native language with reliable information, the participants in this study found the idea very appealing. They were ready to suggest a variety of topics that such an app should contain, including fetus development, changes to their body, nutrition, breastfeeding, clinical and laboratory exams, how to take care of their body, infant care, psychological development, and many more. Identified learning needs appear similar to those described in other studies^3,24,28^. It is worth mentioning that Greek pregnant women expressed their need for more information about natural birth and home birth, which may not be surprising given the very high cesarean rate in Greece which is estimated to be around 50–65%^29^.

### Strengths and limitations

This study has some limitations that need to be considered. The generalization of the results of this qualitative study should be done with caution even if it is expected that qualitative studies do not have any claim regarding generalizability of the findings. In this regard, efforts were made to increase the rigor of the findings through the selection of participants with the maximum variations. However, the vast majority of participants were from the city of Athens. Therefore, the findings may not reflect the needs and experiences of women in regional and rural areas of Greece.

While data saturation was achieved, it is unclear whether theoretical saturation was reached as a bigger and more heterogeneous sample of pregnant women and new mothers may have provided more perspectives. Furthermore, the study aimed at gaining an understanding of the information-seeking experience of Greek pregnant women. A quantitative survey among a representative sample of pregnant women is needed to investigate the level of satisfaction with informational support is needed, while specific learning needs should be further explored in a Delphi survey, which can also involve healthcare providers alongside pregnant women.

## CONCLUSIONS

This qualitative study provides information about the learning needs, the sources of information and the satisfaction with the informational support of Greek pregnant women during the perinatal period. Our study findings conclude that learning needs during pregnancy are different between multiparous and nulliparous pregnant women. Multiparous pregnant women were more worried about the postnatal period, the development and care of the neonatal, while the nulliparous pregnant women seemed to be more worried about the antenatal period, as they have not experienced birth before and they do not know what to expect at labor. Additionally, it was concluded that the content of antenatal classes should be revisited and tailored to address pregnant women’s learning needs more holistically. A major source of information for pregnant women is the Internet as well as other informal sources such as friends and family. Taking into consideration the varied quality of information on the Internet, together with the fact that many pregnant women tend to avoid discussing such information with health professionals, it is vital for health professional to acknowledge this reality and provide pregnant women with trusted websites. It is also particularly important for health professionals to train in communication skills, to expand their knowledge with the latest resources and update their digital literary. Further research is needed to explore healthcare professionals’ attitudes and perceptions of their role in order to determine effective and meaningful ways to incorporate technology and digital resources in clinical and community practice.

## Data Availability

The data supporting this research are available from the authors on reasonable request.
